# Intrahepatic Portosystemic Shunt Causing Hepatic Encephalopathy in a Non-cirrhotic Patient

**DOI:** 10.7759/cureus.91487

**Published:** 2025-09-02

**Authors:** Hailey J Carruthers, Natalie M George, Alexander Eskandarian, Dion Harris, Arif Musa, Ali Harb

**Affiliations:** 1 Department of Radiology, Wayne State University School of Medicine, Detroit, USA; 2 Department of Radiology, Michigan State University College of Osteopathic Medicine, East Lansing, USA

**Keywords:** embolization, hepatic encephalopathy, hyperammonemia, intrahepatic portosystemic shunt, non-cirrhotic, vascular malformation

## Abstract

A 60-year-old woman presented with persistent nausea, new-onset tremulousness, and altered mental status. Abdominal and pelvic computed tomography (CT) revealed a large intrahepatic portosystemic connection, suspicious for a portal venous malformation. She underwent two image-guided embolization procedures. The first, via a transcaval approach, reduced shunt flow and improved ammonia levels. The second, using combined transhepatic and transfemoral access, achieved complete occlusion and led to sustained clinical improvement. This case underscores the importance of considering hepatic encephalopathy in non-cirrhotic patients with altered mental status and recognizing portosystemic shunts as a potential underlying cause.

## Introduction

Intrahepatic portosystemic shunts are abnormal vascular communications between the portal and hepatic veins. They may be congenital, arising from persistent embryologic vessels such as the omphalomesenteric venous system, or acquired, secondary to trauma, prior abdominal surgery, or conditions like portal hypertension [1,2]. However, no single etiological theory has been definitively established [1]. The estimated incidence is 1 in 30,000 to 1 in 50,000 live births [3]. Although often asymptomatic, these shunts can lead to significant complications, most notably hepatic encephalopathy (HE), as large volumes of blood bypass hepatic detoxification. Because of the rarity of the condition, particularly in non-cirrhotic adults, diagnosis is frequently delayed or overlooked. High clinical suspicion is warranted in patients presenting with altered mental status and no evidence of hepatic dysfunction [4,5]. Hyperammonemia may suggest HE, which is initially managed with lactulose therapy. When a portosystemic shunt is identified as the cause, interventional embolization is the treatment of choice. Here, we report a rare case of an intrahepatic portosystemic shunt in a non-cirrhotic adult requiring sequential embolization for definitive management, underscoring the pivotal role of imaging in diagnosis and treatment planning.

## Case presentation

**Figure 1:**
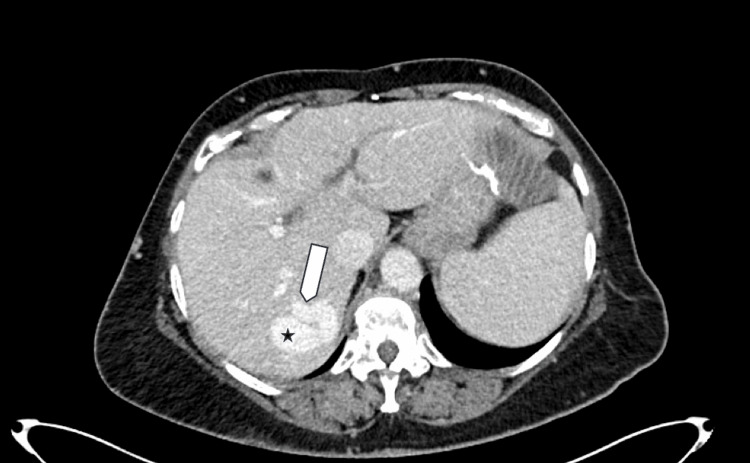
Portal venous-phase axial CT showing a portosystemic shunt The arrow indicates the feeding right portal vein, and the star marks the draining right hepatic vein.

**Figure 2:**
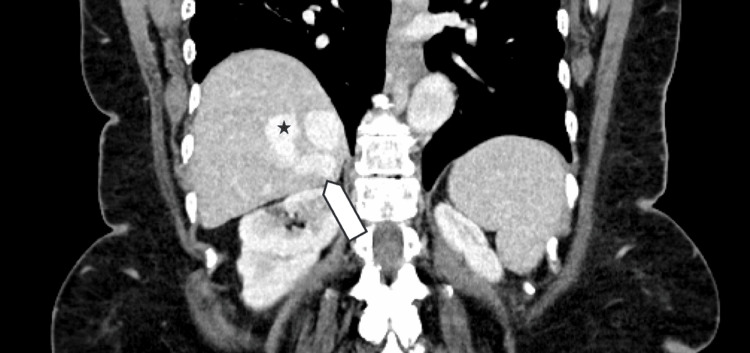
Portal venous-phase coronal CT showing a portosystemic shunt The arrow indicates the feeding right portal vein, and the star marks the draining right hepatic vein.

A 60-year-old woman presented to the emergency department with abdominal pain, nausea, vomiting, and constipation for two days. Her past medical history included peptic ulcer disease, and her surgical history was notable for hemigastrectomy with Billroth II reconstruction, hernia repair, and cholecystectomy. Initial computed tomography (CT) of the abdomen and pelvis demonstrated a partial small bowel obstruction, likely related to postoperative adhesions, and incidentally revealed an intrahepatic portosystemic shunt measuring up to 4 cm in diameter. Imaging showed a communication between the right portal vein and right hepatic vein (Figure 1 and Figure 2). This abnormal vascular connection allows portal venous blood to bypass the hepatic parenchyma and drain directly into the systemic circulation.

The patient was admitted for conservative management, including bowel rest, IV fluids, and opioid analgesics. Her condition improved, and she was discharged with laxatives and outpatient follow-up. However, she returned two days later with new-onset confusion, tremulousness, and persistent nausea. Her serum ammonia level was markedly elevated at 166 µmol/L. Lactulose was initiated, but due to minimal clinical improvement, interventional radiology was consulted.

Under ultrasound guidance, access to the right common femoral vein was obtained, and the right hepatic vein was catheterized. Hepatic venography revealed three suspected efferent vessels. Multiple vascular coils were deployed, which reduced shunt flow. Ammonia levels improved to 111 µmol/L, and a staged intervention was planned. However, the patient discharged herself and was lost to follow-up. 

**Figure 3:**
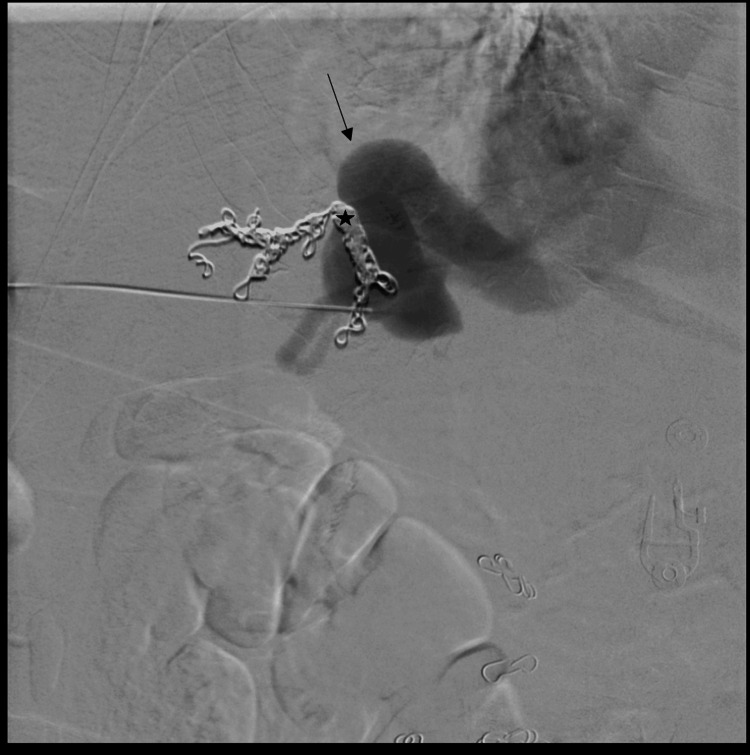
Transhepatic portal venogram showing persistent shunt flow despite prior coil embolization The arrow indicates the portal-systemic communication, and the star denotes the coil mass within the shunt tract. Previously placed vascular coils are visible within the shunt tract. This finding indicates patency of the shunt following coil embolization.

**Figure 4:**
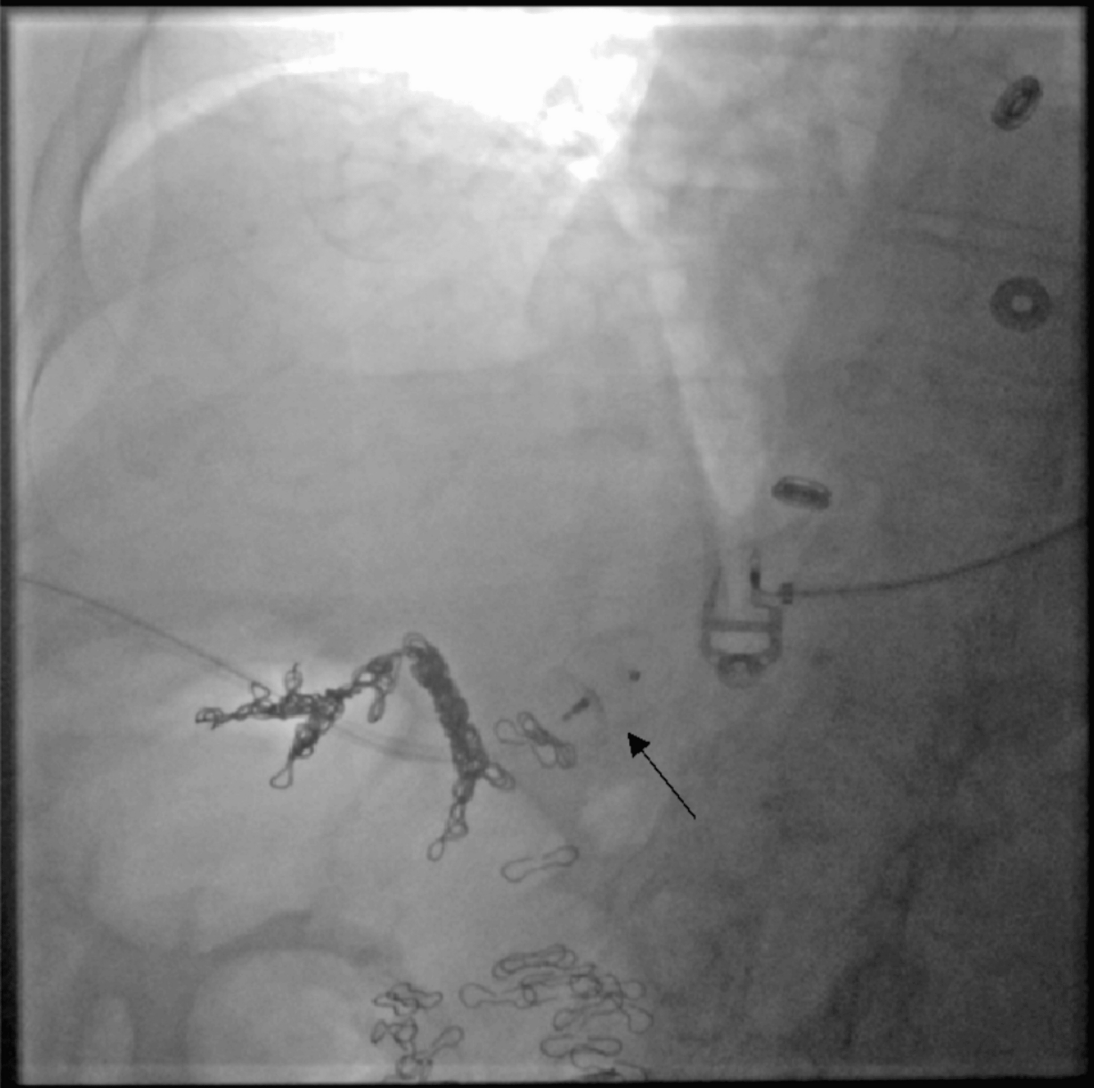
Fluoroscopic image demonstrating deployment of a vascular plug (arrow) via a transhepatic approach, occluding the portal inflow

**Figure 5:**
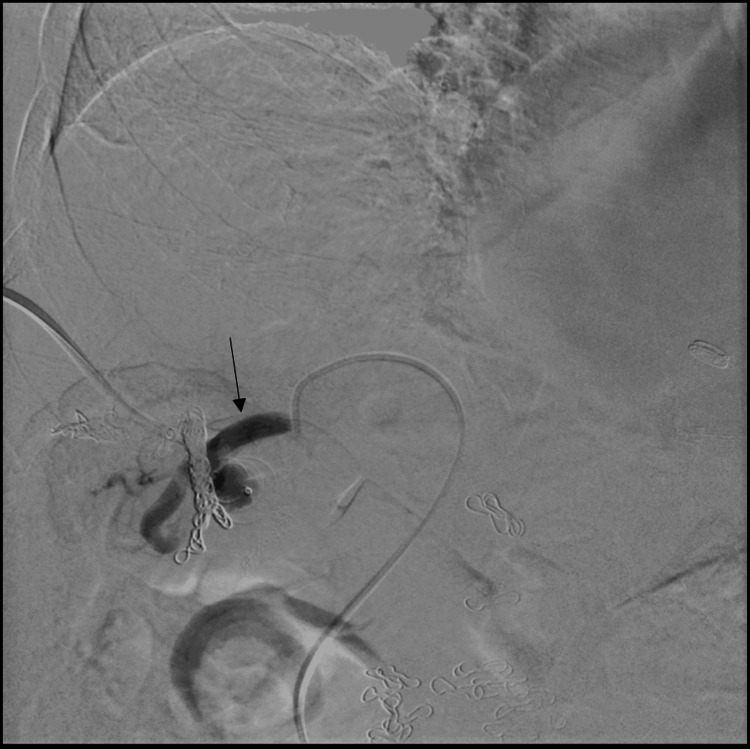
Transfemoral hepatic venogram showing residual shunt flow The arrow indicates the persistent portal-systemic connection despite prior embolization. This persistent flow is visualized despite prior embolization efforts, indicating incomplete occlusion of the shunt.

**Figure 6:**
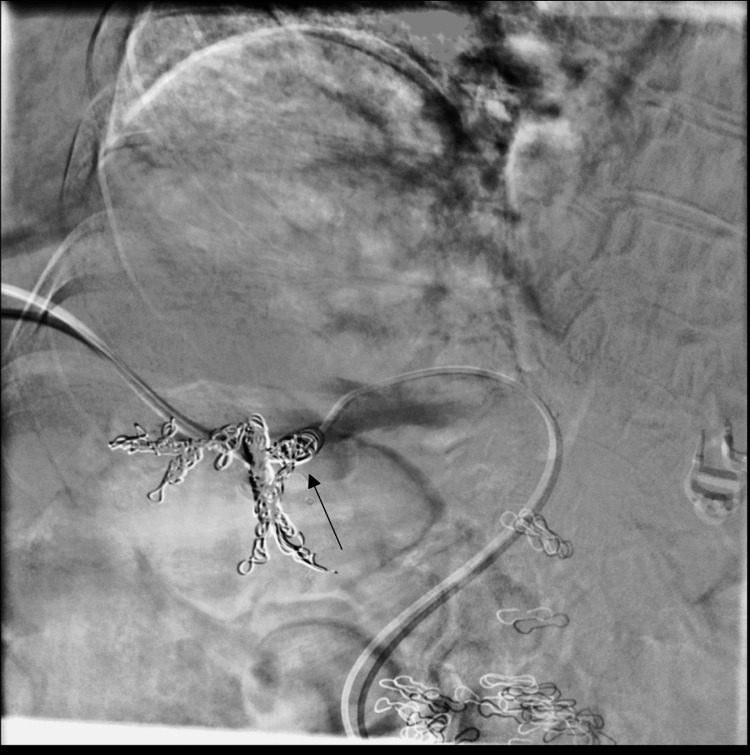
Final hepatic venogram following additional coil embolization and plug placement, demonstrating complete occlusion with no residual flow The arrow indicates the vascular coils within the shunt tract. A vascular plug is also present at the occlusion site, with no residual shunt flow visualized.

Two months later, she returned with recurrent symptoms and an ammonia level of 144 µmol/L. A second embolization was performed using a combined transhepatic and transfemoral approach. A transhepatic portal venogram confirmed persistent shunt flow (Figure 3). Via the transhepatic approach, a 14-mm Amplatzer vascular plug was deployed, effectively occluding the portal inflow (Figure 4). Subsequently, through transfemoral access, the right hepatic vein was catheterized. Venography confirmed the nidus and dilated outflow vessels (Figure 5). Additional vascular coils were deployed, resulting in complete shunt stasis (Figure 6). Final venography demonstrated no residual fistulous connection. Post-procedure, ammonia levels decreased to 67 µmol/L, and the patient was discharged in stable condition with resolution of her encephalopathy.

## Discussion

This case underscores the complexity of diagnosing and managing intrahepatic portosystemic shunts, especially in non-cirrhotic patients. These shunts may remain clinically silent until patients develop acute hepatic encephalopathy. Encephalopathy results from systemic accumulation of neurotoxins, most notably ammonia, which is normally detoxified by the liver. By diverting portal blood away from hepatic metabolism, portosystemic shunts predispose to hyperammonemia and neurologic symptoms. Older adults may be particularly susceptible due to reduced hepatic reserve and age-related cognitive decline. While congenital shunts are thought to arise from persistence of omphalomesenteric vein remnants [6], acquired forms are more often linked to trauma, prior surgery, or portal hypertension [4,7].

Several anatomical subtypes of portosystemic shunts have been described, including single large tubular connections, aneurysmal communications, and diffuse peripheral patterns [1,2]. Management is largely guided by the severity of clinical symptoms. Conservative treatment with lactulose is typically first-line for HE, but persistent or recurrent symptoms warrant interventional therapy [4,7]. Our case represents a peripherally localized shunt, which was well-suited to targeted embolization. Endovascular embolization is a minimally invasive option that can be tailored according to shunt anatomy, vascular accessibility, device availability, and operator preference. In this patient, the initial transcaval approach reduced shunt flow and lowered ammonia levels, but definitive resolution required a second procedure. The subsequent embolization utilized dual access via transhepatic and transfemoral routes, allowing deployment of a vascular plug in combination with multiple vascular coils to achieve complete occlusion. Post-procedural imaging and serial ammonia measurements confirmed technical and clinical success.

This case also underscores the importance of early recognition of HE in non-cirrhotic patients. In the absence of underlying liver disease, HE may be overlooked in the differential diagnosis. Contrast-enhanced CT can identify vascular malformations, while confirmatory venography remains the diagnostic gold standard [8]. With increasing awareness and wider availability of advanced interventional radiology, outcomes in patients with symptomatic portosystemic shunts have improved. Our report contributes to the growing evidence supporting embolization as an effective treatment in non-cirrhotic patients who fail medical therapy [9]. 

## Conclusions

This case highlights the importance of recognizing intrahepatic portosystemic shunts as a potential cause of HE in patients without underlying liver disease. A high index of suspicion, supported by imaging and ammonia levels, is essential for timely diagnosis. Endovascular embolization should be regarded as an effective treatment option, with flexibility in approach and use of multimodal techniques often required for complex vascular anatomy. Early recognition and intervention remain critical to optimizing outcomes in these rare but treatable cases.
